# Phytoassisted synthesis of CuO and Ag–CuO nanocomposite, characterization, chemical sensing of ammonia, degradation of methylene blue

**DOI:** 10.1038/s41598-024-51391-2

**Published:** 2024-01-18

**Authors:** Muhammad Farooq, Shaukat Shujah, Kamran Tahir, Syed Tasleem Hussain, Afaq Ullah Khan, Zainab M. Almarhoon, Khulood Fahad Alabbosh, Abdulaziz A. Alanazi, Talal M. Althagafi, Magdi E. A. Zaki

**Affiliations:** 1 Department of Chemistry, Kohat University of Science and Technology, Kohat, 26000 Pakistan; 2 Institute of Chemical Sciences, Gomal University, D. I. Khan, KP Pakistan; 3 School of Chemistry and Chemical Engineering, Jiangsu University, 301 Xuefu Road, Zhenjiang, 212013 China; 4 Chemistry Department, College of Science, King Saud University, P. O. Box 2455, Riyadh, 11451 Saudi Arabia; 5 Department of Biology, College of Science, University of Hail, Hail, 2440 Saudi Arabia; 6 Department of Chemistry, College of Science and Humanities in Al-Kharj, Prince Sattam Bin Abdulaziz University, Al-Kharj, 11942 Saudi Arabia; 7 Department of Physics, College of Science, Taif University, Taif, 21944 Saudi Arabia; 8 Department of Chemistry, College of Science, Imam Mohammad Ibn Saud Islamic University, Riyadh, 11623 Saudi Arabia

**Keywords:** Biochemistry, Environmental sciences, Chemistry

## Abstract

The elimination of hazardous industrial pollutants from aqueous solutions is an emerging area of scientific research and a worldwide problem. An efficient catalyst, Ag–CuO was synthesized for the degradation of methylene blue, the chemical sensing of ammonia. A simple novel synthetic method was reported in which new plant material *Capparis decidua* was used for the reduction and stabilization of the synthesized nanocatalyst. A Varying amount of Ag was doped into CuO to optimize the best catalyst that met the required objectives. Through this, the Ag–CuO nanocomposite was characterized by XRD, SEM, HR-TEM, EDX, and FTIR techniques. The mechanism of increased catalytic activity with Ag doping involves the formation of charge sink and suppression of drop back probability of charge from conduction to valance band. Herein, 2.7 mol % Ag–CuO exhibited better catalytic activities and it was used through subsequent catalytic experiments. The experimental conditions such as pH, catalyst dose, analyte initial concentration, and contact time were optimized. The as-synthesized nanocomposite demonstrates an excellent degradation efficacy of MB which is 97% at pH 9. More interestingly, the as-synthesized catalyst was successfully applied for the chemical sensing of ammonia even at very low concentrations. The lower limit of detection (LLOD) also called analytic sensitivity was calculated for ammonia sensing and found to be 1.37 ppm.

## Introduction

Water pollution is a worldwide problem faced both by developed and developing countries. In urban areas water is polluted by heavy industrialization while in rural areas it is mostly due to land erosion and mining^1^. Industrial water is saturated with both organic pollutants such as dyes like methylene blue, malachite green, methyl orange, etc. and inorganic pollutants such as heavy metal ions like copper, nickel, chromium, arsenic, lead, etc^2–8^. Water polluted with organic dyes and heavy metals is a serious threat to both land and water systems^6–8^. Release of these specific pollutants into environment causes the damage to mammalian cells, burning of the eyes, lungs irritation, vomiting, nausea, liver tumors, etc.^9–12^. Therefore, water require proper treatment and removal of these organic dyes and heavy metals before its discharge to the ecosystem. Conventional methods include membrane separation, electrolytic process, liquid–liquid extraction, ion exchange, and precipitation as carbonates, sulfates, or hydroxides^13–16^. However, each method has its own advantages and disadvantages depending on its operational cost, working time, ease of experimental setup, chemicals, regeneration, and other overhead expenses^17–21^. Some of the most serious disadvantages that make it unacceptable is incomplete removal, high operational energy, and production of toxic sludge that requires disposal^22^. So, the attention of the researcher is reverting towards adsorption which is economical, speedy, high removal efficiency, simple experimental setup, and applicable at low and high concentration levels^22,23^. Activated carbon is the most common adsorbent used the for removal of both organic and inorganic stuff. However, its high operational cost and lengthy regeneration procedure have limited its use^24,25^. So the use of low-cost agricultural waste such as rice husk, plant leaf powder, etc. is becoming the new trend. However low surface area, low adsorption capacity and prolonged removal time of this biosorbent are unavoidable drawbacks that necessitate further search for adsorbent which is economical, sensitive, nontoxic, and can remove adsorbent in low concentration as well^26,27^.

Various industrious use different synthetic and organic dyes for colouring their products. Methylene blue which is also called basic blue**-**8 is a cationic dye. Its trade names are Urelene blue, Provay blue, Prove blue. It is a heterocyclic organic compound which is frequently used in textile, cosmetics, paper and pharmaceutical industries and biological staining. As medicine this dye can also be used for the treatment of methemoglobinemia but this chemical could not be recommended as medicine because it has very hazardous effects on living things. On inhalation and ingestion this dye can cause permanent injuries in human and in other animals. Methylene blue can cause headache, vomiting, shortness of breath, nausea, diarrhea, high blood pressure, site necrosis (SC), eyes burring effects and abdominal pain in human beings. Among various modern methods, photocatalytic degradation is a unique method for the cationic dyes elimination process^28–30^. Photodegradation is a chemical oxidation reaction catalyzed by nanoparticles that can degrade stable organic dyes like methylene blue. Amongst them, ZnO is a well-known photocatalyst that has been explored deeply for its catalytic and adsorption potential but CuO is less common, having all the catalytic properties found in typical metal oxide nanoparticles^31–33^. CuO is a p-type semiconducting material having a bulk band gap of 1.2 eV which makes it applicable in solar systems, battery anodes, nanofluids, superconducting materials, gas sensors, transistors, magnetic storage devices, lithium-ion batteries, ceramic resistors, IR filters and recently in formation of solid-fuel for rocket vehicles^34^. All these applications can be dramatically enhanced by doping of transition and non-transition metals which improve their electrical properties by changing the band gap. Mohamed Basith et al. have doped Ni and Fe and observed that band gap width increases up to 4.3 eV^35,36^. Doping of Ag metal can modify the catalytic and semiconducting properties even better due to due to its versatile metallic nature. Currently, only a few articles are presents that show the doping of Ag to CuO. In this context Jichun Huang et al. have investigated that Ag doping to CuO can enhance the electrical conductivity of Ag covered CuO nanosheet arrays^37^. Hooch Anting et al. studied the successful doping of Ag to CuO and applied it for hydrogen sensing^38^. Nasrin Ghasemi et al. examined the improvement in antibacterial properties by comparing CuO and Ag doped CuO^39^. Jianbo Yang et al. synthesized Ag–CuO composite nanosheets and investigated the improvement in photocatalytic properties by degrading methyl orange^40^.

Ammonia is one of the toxic chemical species that is readily soluble in water producing ammonium ion. In addition to other uses like paper, plastic, textiles, explosives, pesticides, refrigeration, cleaning products, and yes about 90% of ammonia is used in the fertilizer industry^41^. In air, its amount as low as 5 ppm can be recognized by odor. Therefore, the risk of ammonia toxicology to animals is higher as compared to other gases. The adverse effect of ammonia includes irritation of the eyes, nasal, throat, and chest, cough, edema of the throat, shock, restlessness, cyanosis etc.^42^. One of the unique properties of nanoparticles is its successful application in chemical sensing for the presence of various pollutant such as CO, SO_2_, NO, N_2_O, NO_2_ etc. in wastewater^43,44^. Metal oxide-based chemical sensors are extensively used due to its low cost, easy operation, nontoxic and stability. However great deal of research is in progress to improve its sensitivity, selectivity, and response time^45,46^. Compare to other metal oxide, ZnO has been extensively used as a chemical sensor. For example, Ahmed et. al. used it for the detection of O_2_ gas^47^, Tan et al. for the detection of CO^48^, Ismail et. al. the for detection of N_2_H_4_^49^, Ganbavle et. the al. for detection of NO_2_^50^, Leidinger et. al. for detection of volatile organic compounds (VOCs) etc.^51^. Some other metal oxides like SrO, TiO_2_, Al_2_O_3_, WO_3_, SnO_2_, RuO_2_ were also reported as chemical sensors^52,53^. However, very limited work has been published on the chemical sensitivity of CuO. Doping of Ag to CuO can bring even better results due to improvement in electrical properties.

Biological methods also called green methods are ecofriendly, economical single step process that generates a nontoxic product that can be handled easily. In biological methods phytochemicals present in plant extract are used for reduction and stabilization^54^. A. Tresa Babu et al. synthesized Ag-doped CuO in the presence of *Sida rhombifolia* leaf extract and explored its catalytic properties by degradation of stable aromatic dyes^55^. Maruthupanday et. al. prepared CuO nanoparticles using *Camellia japonica* plant extract as a reducing agent and successfully applied it for the optical sensing of various metal ions^56^. Plants encompass diverse secondary metabolites, including alkaloids, flavonoids, saponins, and steroids, which serve crucial functions as both reducing and stabilizing agents during the synthesis of nanoparticles. Flavonoids and saponins have been documented as effective capping agents in regulating the rate of nanostructure formation, mitigating agglomeration tendencies^57–61^.

In the present article, we have reported the synthesis of Ag-doped CuO (Ag–CuO) in the presence of *Capparis decidua* plant extract to reduce the metal ion and stabilize the product. Many scientific reports show synthesis of Ag–CuO, however most of them adopt chemical synthesis route which have serious impacts on environment. Some synthetic method also followed use of phytochemical present in plant extract, however no work has been published on this reducing phytochemical rich plant, *C. decidua* plant. Additionally, in reported work, the nanocomposites have solid compact physical state due to which they have low surface area, hence their catalytic activities are significantly reduced. In present work, the preparation method is slightly modified so that Ag–CuO have some porous architecture due to evolution of residual gases during calcination. Due to this porosity surface area and catalytic properties are significantly enhanced as compared with the reported work. Different amount of Ag was doped to optimize the product that is most effective in photocatalytic degradation, chemical sensing, and adsorption process. Furthermore, a numerous characterization technique such as XRD, SEM, EDX, HRTEM, and FTIR were used to measure particle size, shape and bond formation in CuO nanoparticles. Photocatalytic properties of Ag–CuO were examined by degrading methylene blue dye. The as synthesized nanomaterial was applied for the chemical sensing of ammonia in an aqueous solution.

## Experimental

### Chemicals and materials

The branches of the *C. decidua* plant were collected from the hilly part of Kohat University Pakistan. All the chemicals such as hydrochloric acid, nitric acid, copper sulfate, copper nitrate, lead chloride, lead acetate, ammonium hydroxide, sodium hydroxide, and methylene blue were obtained from Sigma Aldrich Pakistan and used directly without further treatment. A Stock solution of Pb(II) ion was prepared by dissolving a calculated amount of salt in deionized water. A fresh solution of methylene blue and ammonia was prepared in deionized water at the time of use. All the glassware was purchased from Sigma Aldrich Pakistan. The glassware was washed and preheated every time before use.

### Synthesis of nanomaterial

#### Preparation of plant extract

The plant material has complied with the approval of the Department of Botany, Kohat University of Science and Technology, Pakistan, with national, and international guidelines and legislation. The branches of the wild *C. decidua* plant were collected from the suburbs of Lakki Marwat, Pakistan. It was washed with tape water many times and then rinsed with deionized water and soaked at room temperature. Shaded dried branches of the *C. decidua* plant were powdered and 5 g of it was stirred with 100 mL of deionized water. After 2 h of stirring at 60 °C it was filtered and the filtrate was kept in cold storage for further use.

#### Synthesis of CuO

Calculated amount of copper sulphate was dissolved in 50 mL deionized water and stirred for 20 min. Specific amount of plant extract (20 mL) was added drop wise with constant stirring at 60 °C. After 2 h of stirring the pH was adjusted at 10 with freshly prepared ammonia solution and again stirred for 2 h. Solution was kept in teflon autoclave for 5 h. After that it was filtered and the black mass obtained was dried in oven at 100 °C. It was placed for about 3 h in furnace at 400 °C for calcination. The calcined CuO was grinded and stored in plastic viols.

#### Synthesis of CuO

Calculated amount of silver nitrate was dissolved in 50 mL deionized water and stirred for about 10 min. 20 mL of plant extract was added drop wise with constant stirring and temperature was maintained at 60 °C. During this procedure pH drops significantly which is adjusted at 9 by adding freshly prepared ammonia solution and again stirred for 2 h. Highly basic pH must be avoided to restrict formation of Oxides and hydroxides. The colour of solution changed to yellow then it was kept in teflon autoclave for 5 h. After that it was filtered and the grey mass obtained was dried in oven at 120 °C. It was grinded and stored in plastic viols.

#### Synthesis of Ag–CuO

1 g of calcined CuO was added to 20 mL deionized water and sonicated for 10 min. 50 mL of deionized water was added and stirred for 30 min. To this solution 0.02, 0.04, 0.06, 0.08 and 0.1 g of AgNO_3_ was added to prepare 0.92%, 1.8%, 2.7%, 3.6% and 4.4% Ag–CuO solution respectively. The percentage of Ag doping was calculated using Eq. (1). Plant extract was added and the solution was continuously stirred. pH was adjusted at 10–11 by adding freshly prepared ammonia solution and stirred for further 2 h. After that it was filtered, dried in oven and the dried mass was Ag–CuO.

1 $$\%\, mol\, of\, Ag= \frac{n\, of\, AgN{O}_{3}}{n\, of\, AgN{O}_{3}+n\, of\, CuO} \times 100$$

where n is number of moles.

### Characterization

Synthesized CuO and Ag–CuO were characterized through different techniques. XRD spectra were obtained in the range of 20°–80° to check the crystallinity of the synthesized nanomaterials. Average particle size was calculated using the Scherrer equation. SEM images were obtained to find information about surface morphology, porosity, and surface element distribution. HR-TEM images were studied to obtain crystallite shape, size, and distribution of Ag particles into CuO lattices. EDX peaks were analyzed to check the purity and presence of constituent elements in the nanomaterials. FT-IR spectra was obtained to confirm the formation of metal–oxygen bond and functional groups presents in plant extract and synthesized nanomaterials.

### Degradation of MB

The photocatalytic potential of the CuO and Ag–CuO were investigated by degradation of methylene blue (MB). Experimentally, solutions of MB with different concentrations were prepared. The specific amount of the synthesized photocatalyst with different Ag doping, was added and stirred in the batch-type reactor under UV visible light illumination till the equilibrium. A bulb ((λ ≥ 420 nm, HPL-N, 125 W, Philips, China) was used to supply visible light irradiation. 5 mL of the mixture was taken out and centrifuged. It was filtered and the dye remaining concentration in the filtrate was determined by checking the absorbance spectrophotometrically. MB has an absorbance band in the visible region at 665 nm. The blue shift in the peak with time confirm the degradation and removal of MB by the photocatalyst. The effect of various parameters such as Ag doping, pH, catalyst amount, contact time, and dye initial concentration were studied to optimize the experimental condition for maximum removal. The percent degradation of MB was calculated by Eq. (2).

2 $$\%\, Removal=\frac{{C}_{o}-{C}_{e}}{{C}_{o}}\times 100$$

where (C_o_) is the initial while (C_e_) the is final concentration of the analyte.

### Chemical sensing of ammonia

Chemical sensing capabilities of 2.7 mol % Ag–CuO were explored against ammonia solution. Experimentally ammonia solutions of 0, 5, 10, 20, 30, and 40 ppm were prepared. 2 mL of the solution was taken and a specific amount of the catalyst was mixed with it. To check the sensing potential its absorbance was measured by UV visible spectrophotometer.

## Results and discussion

### Characterization

#### XRD results

**Figure 1:**
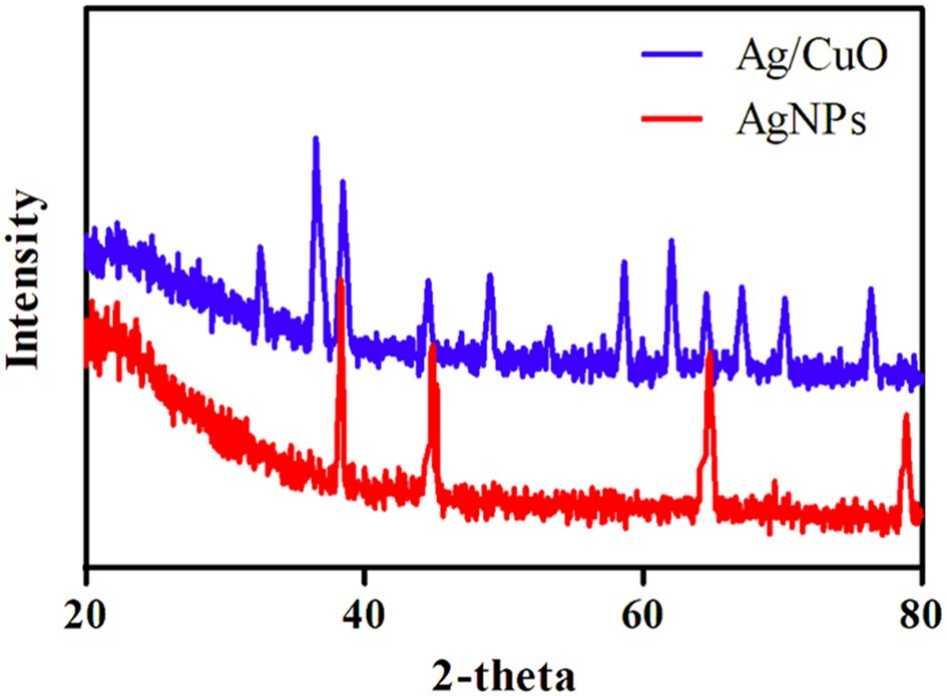
XRD diffractogram of Ag–CuO and Ag nanoparticles.

The XRD spectra of CuO and 2.7 mol % Ag–CuO are presented in Fig. 1. XRD spectra of pure CuO consist of diffraction peaks at 2 $\theta$ range of 32.64, 35.74, 38.97, 48.84, 53.32, 58.54, 61.42 and 66.73 represents the planes with indices (110), (111), (200), (− 202), (020), (202), (− 113) and (022) respectively. The presence of these sharp peaks confirms the monoclinic crystalline structure of CuO. XRD pattern of Ag–CuO shows additional peaks in the region of 2 $\theta =38.87$ and 44.63 which represents (1 1 1) and (2 0 0) planes of Ag. The presence of these additional peaks confirms successful doping of Ag to CuO. The particle size was calculated using Debye Scherrer Eq. (4). Average crystallite size was found to be 21.46 and 18.32 nm for CuO and Ag respectively, which also concede with SEM and TEM results.

4 $$D= \frac{k\uplambda }{\beta cos\theta }$$

here (D) represents nanoparticle size, (k = 0.9) is constant, ($\uplambda )$ is the Cu Kα radiation wavelength, ($\beta )$ is the peak width measured at half maximum intensity and ($\theta )$ is the peak position at 2$\theta$ scale.

#### SEM results

**Figure 2:**
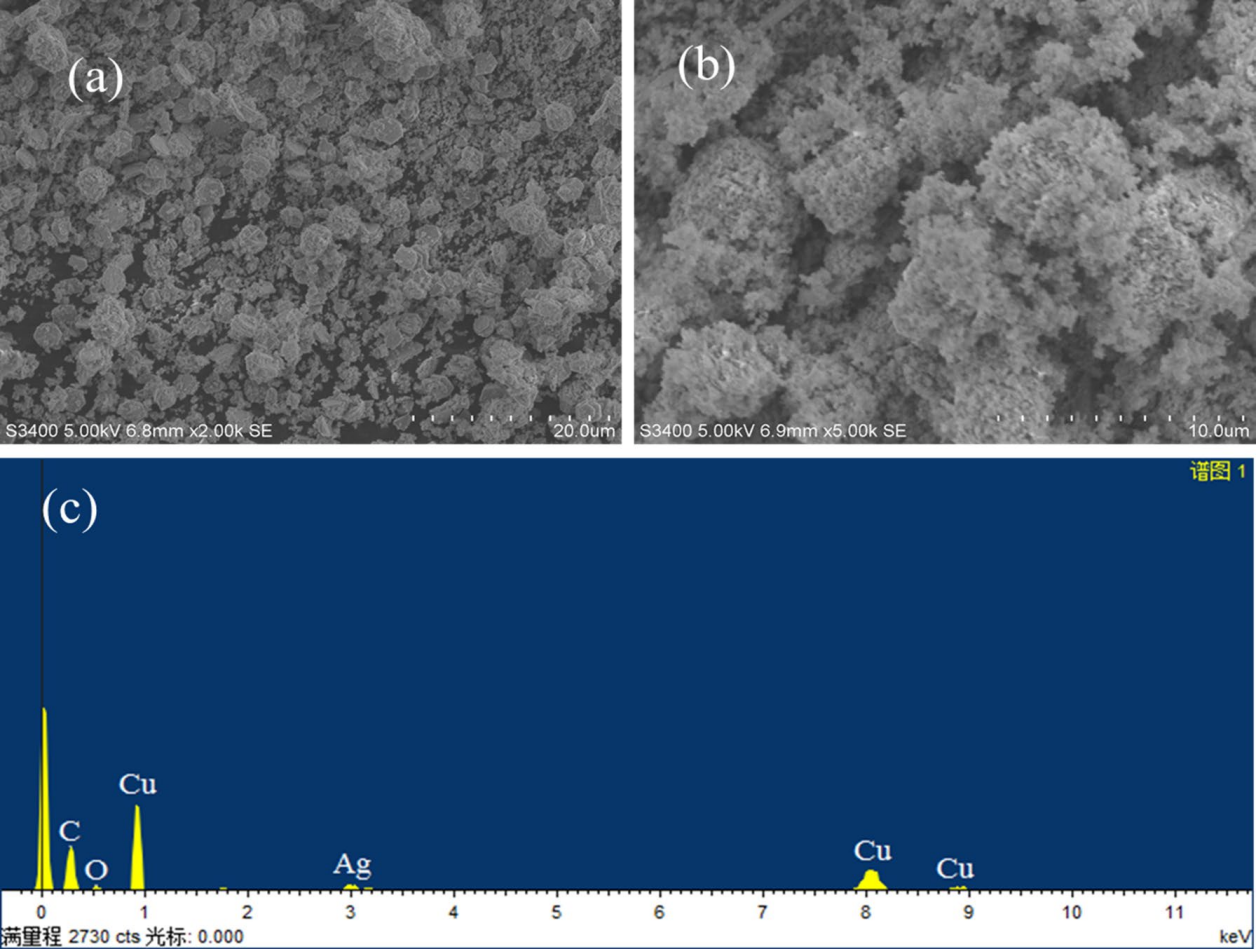
SEM image of (**a**) CuO (**b**) Ag–CuO and (**c**) EDX of Ag–CuO.

Surface morphologies and particle shapes are deduced from SEM images as shown in Fig. 2a for CuO and Fig. 2b for 2.7 mol % Ag–CuO. Figure 2a clearly shows that CuO possesses spherical and rod-shaped particles. On doping Ag to CuO surface shape is modified to a sponge-like porous architecture that possesses greater surface area as shown in Fig. 2b. Doping may increase the inter-particle distance which creates micropores that result in enhancement of catalytic properties. The evolution of exhaust gases at high temperatures may also be one of the reasons of pores creation.

### EDX results

The purity of the sample and the resultant elemental composition of the synthesized, Ag–CuO were checked using EDX spectra as represented by Fig. 2c. EDX spectra of Ag–CuO consist of peaks that are representative of only Cu, O, and Ag. No additional peaks other than the expected ones were observed which confirm the purity of the nanomaterials. Presence of Ag peak also confirm the successful doping to the CuO lattices.

#### TEM results

**Figure 3:**
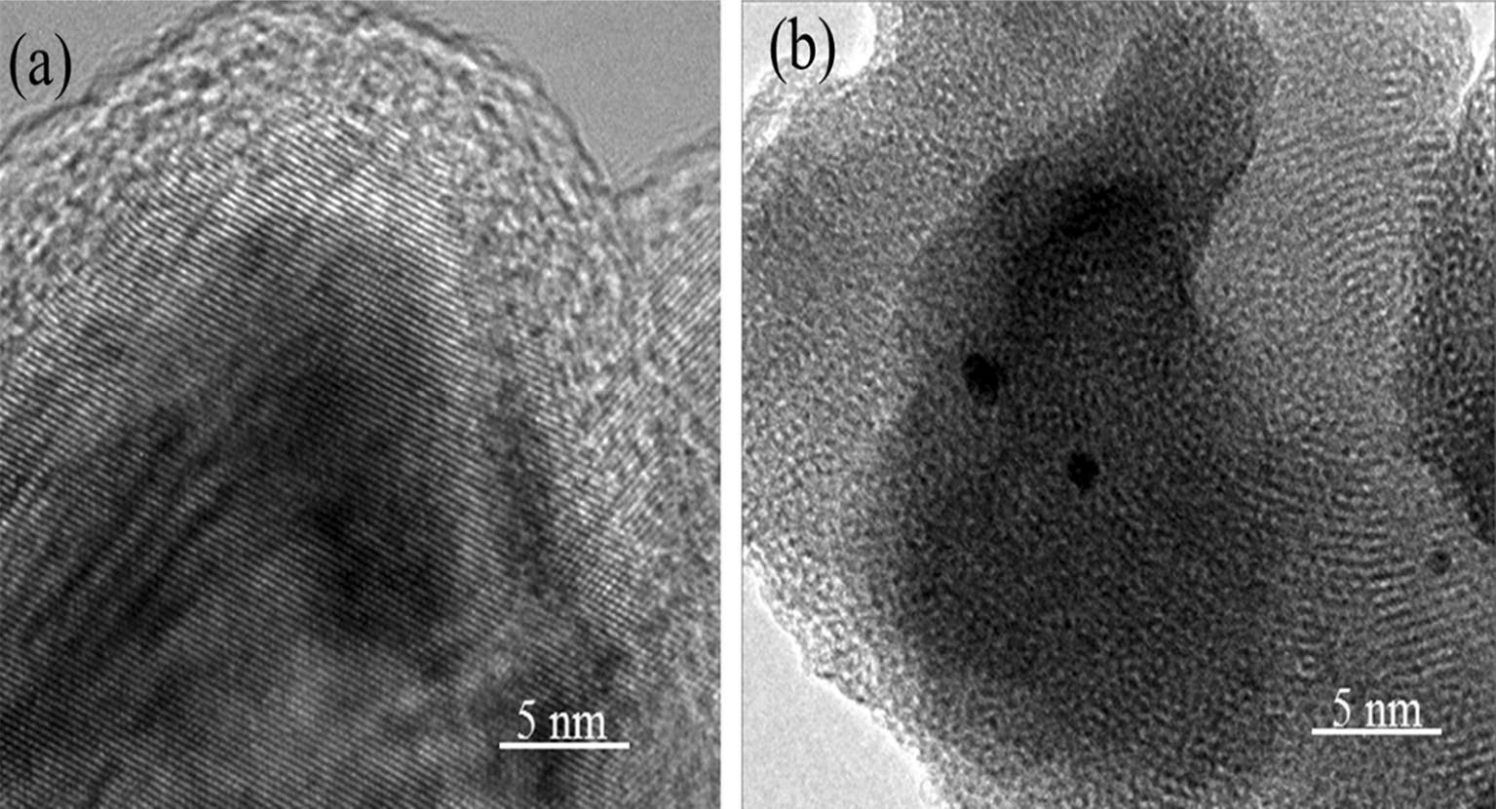
HRTEM image of (**a**) CuO (**b**) Ag–CuO.

Morphologies of crystalline nanomaterials and the crystallite size were confirmed by evaluating HRTEM metaphors of CuO and Ag–CuO as shown in Fig. 3a,b respectively. HRTEM images clarify that Ag is uniformly located on the planes of CuO. Lattice fringes with a spacing of 2.84 A were manifested at a higher magnification level which illustrates the (111) plane of Ag. The average crystallite size lies in the range of 21 nm as manifested by HRTEM is also in accordance with XRD results. Both HRTEM and SEM images show porous sponge like morphologies for Ag–CuO.

#### FTIR results

**Figure 4:**
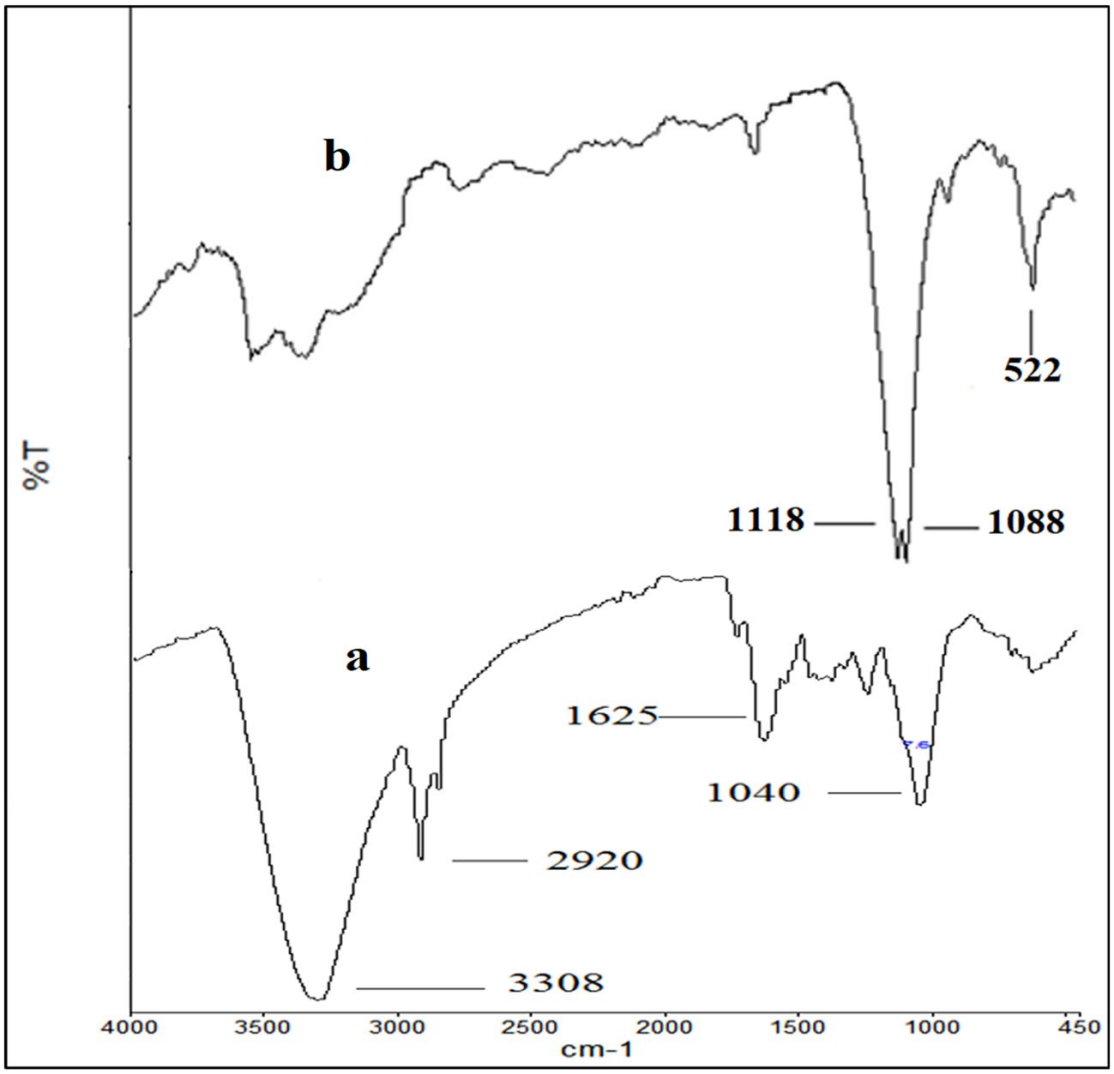
FTIR spectra of (**a**) Plant extract (**b**) Ag–CuO.

Various functional groups present in the phytochemicals of plant extract that are necessary for reduction and stabilization were confirmed by FTIR spectra of plant extract as given in Fig. 4a. Additionally, the formation of copper-oxygen bonds is best illustrated by FTIR studies. Broad bands at 3308 cm^−1^ are allotted to hydroxyl groups from plant sources and water. The band at 1623 cm^−1^ also manifests the presence of a hydroxyl group. The bands due to bending and stretching vibrations of the C–H bond are present in the region of 1040 and 2920 cm^−1^. The absence of a hydroxyl group in the spectra of CuO and the appearance of new bands in the fingerprint region confirm the formation of a copper-oxygen bond, as displayed in Fig. 4b. Metal–oxygen (Cu–O) bond formation is shown by a band at 522 cm^−1^. C–O bond stretching modes give bands at 1116 cm^−1^ and 1432 cm^−1^ as depicted in CuO spectra Fig. 4b.

### Degradation of methylene blue

#### Effect of Ag doping concentration

**Figure 5:**
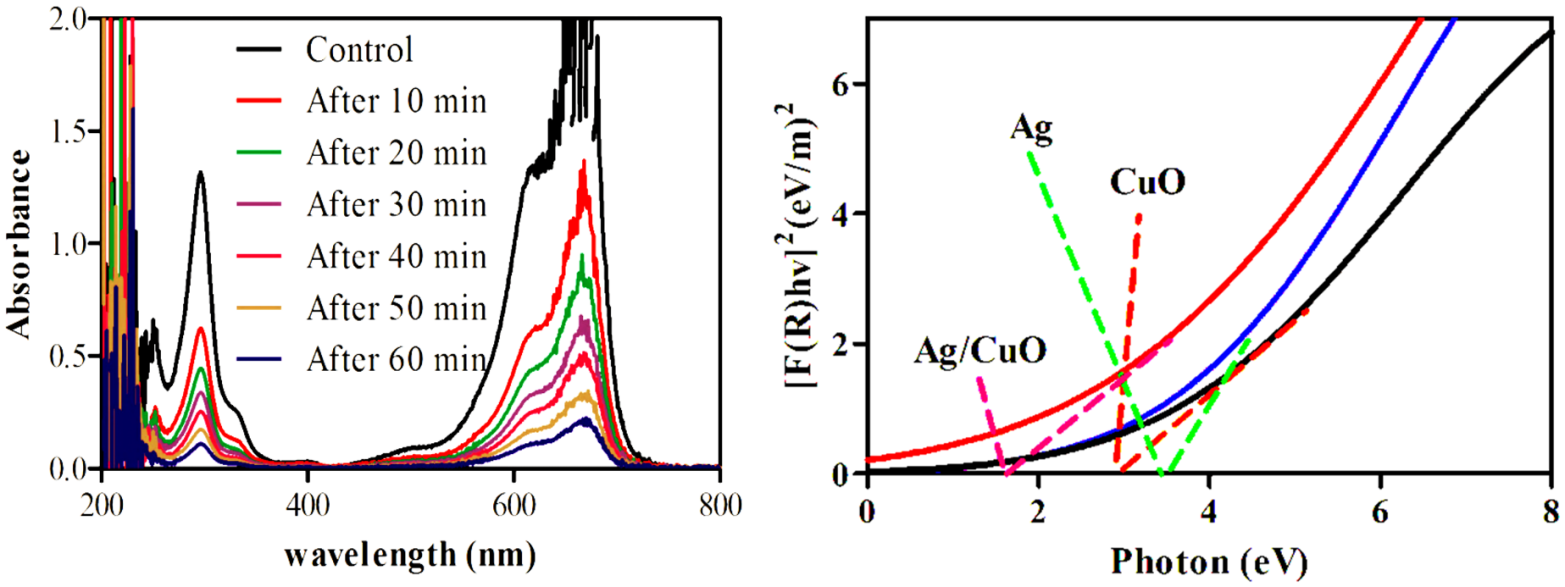
(**A**) UV/Visible spectra of degradation of methylene blue by Ag–CuO, (**B**) Band gap energies comparison of Ag, CuO, Ag/CuO.

**Figure 6:**
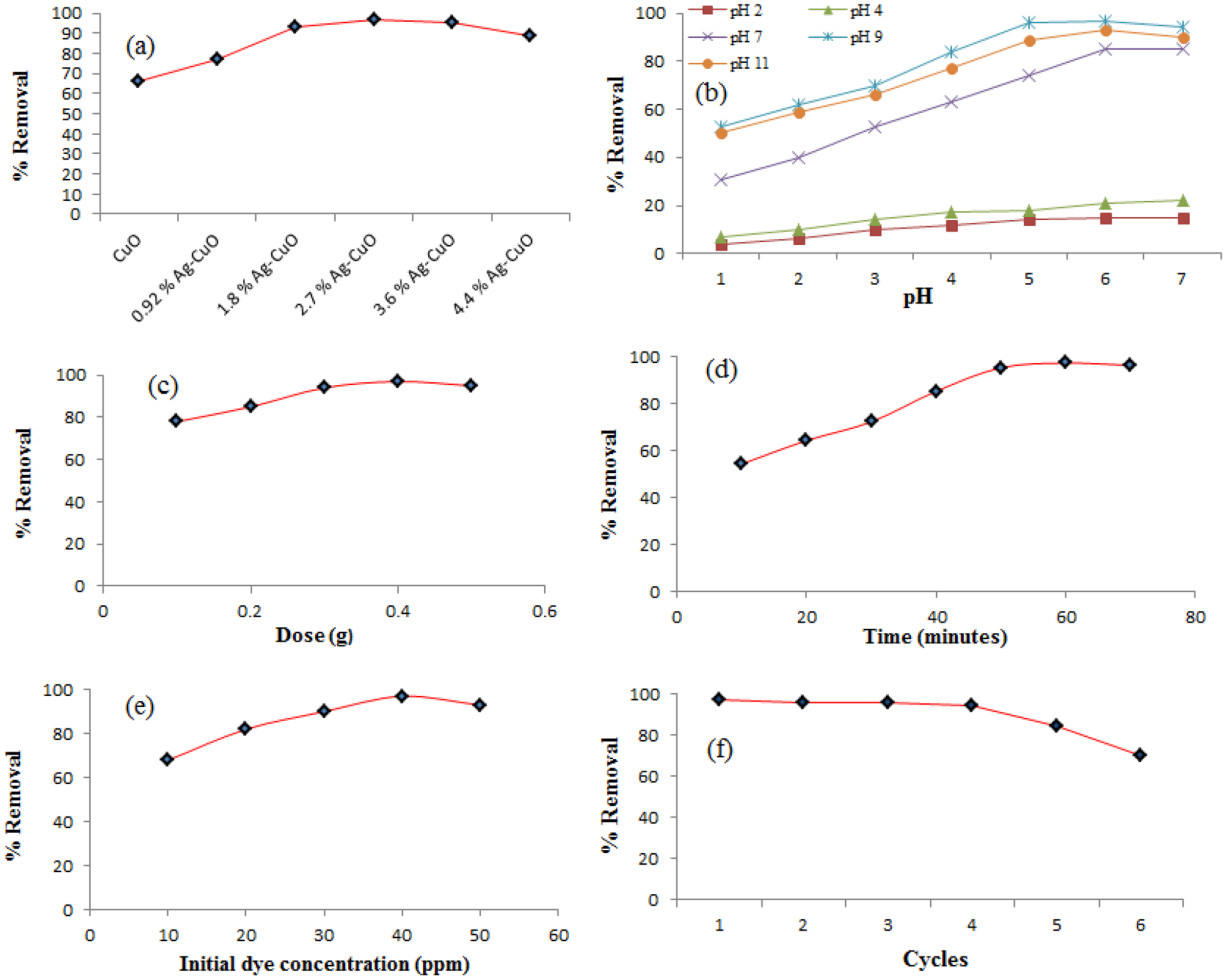
(**a**) Effect of Ag doping, (**b**) pH (**c**) illumination time (**d**) catalyst loading and (**e**) dye initial concentration (**f**) Recyclability study.

To select the photocatalyst with the best activity, the degradation potential of CuO and 0.92, 1.8, 2.7, 3.6, and 4.4 mol% Ag–CuO were examined against methylene blue (MB). 40 ppm solution of MB was taken in batch type reactor and each time a fresh amount (0.4 g/L) of photocatalyst was added under visible irradiation results are depicted by its UV spectra in Fig. 5. In particular, 2.7 mol% Ag–CuO was found best photocatalyst as its activity extends up to 97% as shown in Fig. 6a. Further doping up to 4.4% has a negative effect on photocatalytic activity. Ag doping to CuO enlarges the band gap between the valance and conduction band so the dropback probability of photo-excited electron decreases as Ag is a good sink for a negative charge. Consequently, Ag doping stops the recombination of conduction electrons and positive holes in the valance band which are primary elements responsible for the degradation mechanism^62^. Additionally, doping replaces Cu^2+^ ion with Ag^1+^ ion in the crystal lattice of CuO so due to charge difference oxygen vacancy is created that promotes the photocatalytic activity^63^. When Ag concentration exceeds its upper limits it agglomerates on the surface and creates a recombination center which demolishes the degradation potential^64^. The upper limit of Ag doping concentration with the best activity is not fixed and it may vary for different metal oxides. Method of preparation of the photocatalyst also affects Ag doping concentration^65^.

The Band gap energy of the said nanomaterials were also examined as shown in Fig. 5B. The band gap energies of Ag, CuO and Ag/CuO were 2.8, 2.5 and 1.7 eV, respectively. The smaller band gap energy of Ag/CuO may be due to their synergistic effect.

#### Effect of pH

Adsorbent surface chemical nature is greatly affected by the pH of the medium. The effect of pH was examined in the range of 2–11 on 40 ppm MB solution using 0.4 g of 2.7 mol % Ag–CuO under visible light irradiation at room temperature. Maximum efficiency was observed at pH 9 as shown in Fig. 6b. Generally, pH affects the formation of acid–base equilibrium on the surface of the catalyst during adsorption^66^. The pH of medium reflects the surface charge and so the concentration of H^1+^ and OH^1−^ ions. The surface charge of the material is adjusted by pH change such that it attracts opposite charge species due to electrostatic interactions for maximum removal. As MB is a cationic dye with a positive charge and the catalyst surface becomes negatively charged in the basic medium adsorbate-adsorbent interactions increase and so does the degradation^67^.

#### Effect of catalyst dose

The effect of catalyst dose was studied in the range of 0.1–0.5 using 2.7 mol % Ag–CuO and results are displayed in Fig. 6c. Results show that degradation efficiency increases with an increase in catalyst dose and reaches up to 97% at 0.4 g of catalyst and then decreases. Adsorption is a surface phenomenon that increases with the increase in surface area and amount of the catalyst. As the catalytic amount increases the number of reactive species like photo-excited electrons and oxygen free radicals in the conduction band while positive holes and hydroxyl free radicals in valance band also increases. Combine effect of these two factors results in enhancement of photodegradation ability^68^. Catalytic loading beyond its ideal value results in surface area reduction due to the agglomeration of particles. Also at higher catalytic doses, the solution becomes concentrated, turbid, and opaque so it scatters light, and thus the penetration of visible light irradiation through the solution body is prohibited to reduce photocatalytic degradation^69^.

#### Effect of contact time

The effect of contact time was examined in the range of 10–80 min for 40 ppm MB solution at pH 9 using 2.7 mol % Ag–CuO as a photocatalyst. Degradation/removal of MB increases directly with an increase in time interval up to the equilibrium. When the equilibrium is established degradation becomes constant with time interval. The equilibrium time for Ag–CuO was observed to be 80 min as shown in Fig. 6d. As time interval increases illumination time increases which excites a greater number of electrons so the number of positive holes and other reactive species also increases which enhances the degradation efficiency^70^.

#### Effect of initial concentration of dye

Dye initial concentration was varied from 10 to 50 ppm keeping all other parameters constant. It was observed that initially degradation increases with dye concentration then it becomes constant and finally decreases as shown in Fig. 6e. Maximum adsorption was found for 40 ppm initial concentration. When dye initial concentration increases its adsorption increases so the catalyst uncover/bare surface decreases. Therefore, adsorption of OH^1−^ ion decrease on the catalyst, and as a result production of HO⋅free radical decreases so degradation also decreases^71^. A high concentration of dye may also shelter the catalyst surface from visible light irradiation so fever number of photons strike the surface and the generation of photo-excited electrons decreases. Consequently, the number of reactive species like hydroxyl and oxygen free radical decreases that affect the degradation efficiency^72^.

#### Photodegradation mechanism of methylene blue

Ag doping to CuO acts as an electron scavenger. Electrons are excited under visible light irradiation from the valance band of CuO to its valance band creating a positive hole in the valance band^73^. However due wide band gap this process is slow and requires high energy. This problem can be solved efficiently by Ag doping because the fermi level of Ag is lying at a lower level than CuO so a Schottky junction is created that helps electron to jump in multilevel. These photo-excited electrons in the conduction band are captured by Ag^1+^ ion so their dropback probability is minimized. Surface oxygen reacts with electrons in the conduction band in converted to oxygen free radical (⋅O_2_). Meanwhile in the valance band positive hole reacts with OH^1−^ from water or base and converts it to hydroxyl free radical (⋅OH). Ag^1+^ in the conduction band also create hydroxyl free radical by reaction with OH^1−^. These reactive species (⋅O_2_ and ⋅OH) are unstable, short-lived, strong oxidizing agents that initiate photocatalytic oxidation of tested dye^74,75^.

**Figure 7:**
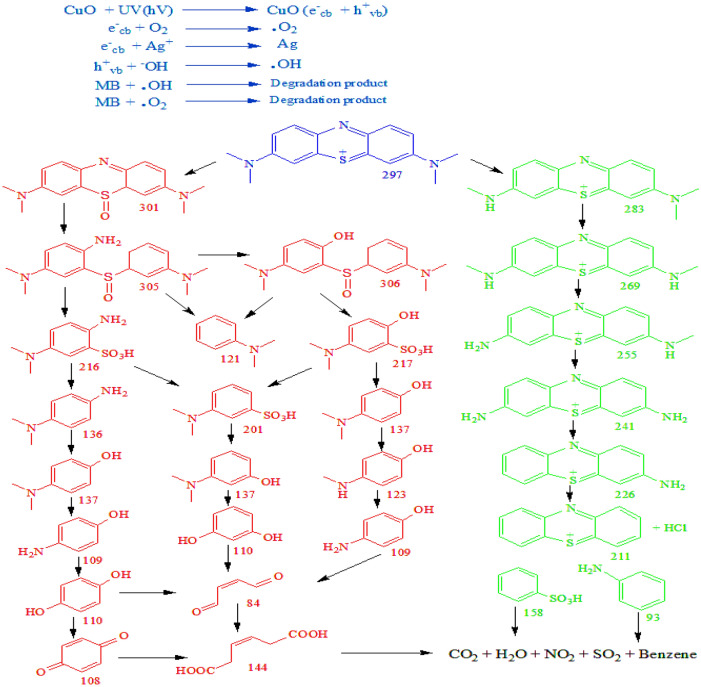
Degradation mechanism of MB.

Photocatalytic oxidation of MB occurs in many redox reactions, producing many intermediate species that were confirmed by GC-MSD. During degradation blue color of MB fades with time and a hypochromic shift occurs in its UV band at 665 nm as shown in Fig. 5. Actually, MB has many auxochromes in its structure that break during the degradation process^76^. Analysis of degradation products suggests two pathways for the degradation mechanism. One way, which is shown by the red color in Fig. 7, involves the breaking of MB through –N=C and –S=C auxochromes in the middle of two benzene rings. This pathway first generates sulfoxide compounds which break into monosubstituted benzene^77^. Another pathway, as shown by the green color in Fig. 7, consists of N-demethylation of two-sided N, N-dimethyl amine groups in MB^78^. This pathway produce, one heterocyclic three benzene ring product that further breaks down CO_2_ and H_2_O^79^.

#### Recyclability study

One of the important parameters that affect stability, efficiency, and consequently cost is the recyclability of the synthesized adsorbent. 0.1 M HCl was used to regenerate the adsorbent. There was very little difference in the degradation capacity in the first four cycles and then it faded off gradually. The slight decrease in adsorption capacity may be due to the penetration of some MB ions into the pores and thus pore diffusion or intraparticle diffusion occur. The results are displayed graphically in Fig. 6f.

#### Comparison of the degradation studies

Table 1 compares the degradation efficacy of the synthetic adsorbent with the usual adsorbents previously described in the literature. It is challenging to select the most effective one because the adsorption capacity depends on the type of adsorbent, the initial concentration of the adsorbate, the preparation method, the cost, etc. The synthesized adsorbent is evaluated against nanomaterials based on CuO, as well as against other metal oxides, and their composites.

**Table 1:** Comparative studies of photodegradation performance of Ag–CuO nanomaterials.

Catalyst	pH	Irradiation time (min)	% Degradation	References
Ag–CuO	9	70	92	^40^
CuO	9	120	63.44	^80^
Ag–CuO	9	60	94.43	^81^
CuO	10	50	90.54	^82^
6Ni–4Cr/TiO	9	90	95.60	^83^
CuO–TiS_2_	10	180	83.18	^84^
CuCr2O4/CuO	10	35	90.00	^85^
Ag–CuO	9	80	97	Present work

### Chemical sensing of ammonia

**Figure 8:**
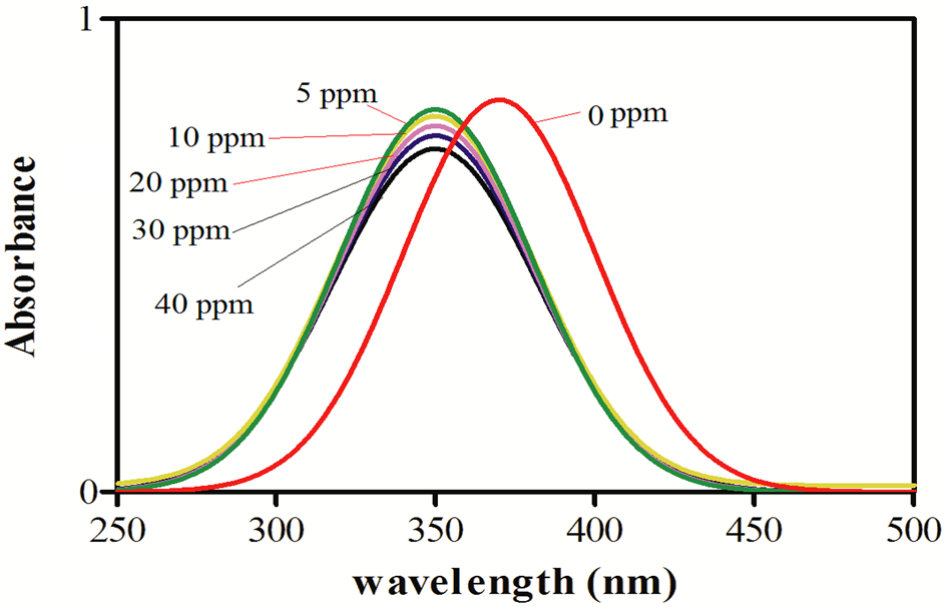
UV/visible spectra of chemical sensing of ammonia by Ag–CuO.

Ag–CuO was used for chemical sensing of an aqueous solution of ammonia having a concentration in the range of 0–40 ppm and the results are displayed in Fig. 8. As shown in the figure the peak at 350 nm which is due to the surface plasmon resonance (SPRs). In metal nanoparticles, SPRs have an intense and broad absorption band that arises due to the coherent oscillation of electrons in the conduction region near the surface of metal nanoparticles. These SPRs have a critical role in chemical and biological sensing. The blue shift was observed by increasing the ammonia concentration as indicated by a new peak at 310 nm. Changes in the inter-particle distance are usually accountable for this change in SPR shift. So, analysis of absorption spectra is a powerful tool for detecting and calculating the concentration of ammonia solution. The new peak at 310 nm is due to the formation of a coordination complex between ammonia and Ag–CuO nanocomposites. The increasing ammonia concentration from 0 to 40 ppm was monitored by the shift in SPRs position and amplitude in UV–visible spectra^86^. Absorbance data was analyzed to find lower limit of detection (LLOD). It is the smallest amount of analyte that can be detected. LLOD is also called analytic sensitivity. Absorbance data was used to find slope of the graph. Form the data standard error intercept (SE intercept) was find. Standard deviation (SD) intercept was calculated by equation (SD = SE × √N) where N is number of sample. From SD, LLOD is calculated by equation (LLOD = 3 × SD/slope) and found 1.37 ppm.

## Conclusion

An efficient nanocatalyst, Ag–CuO was prepared by eco-friendly using *C. decidua* plant material as a natural reducing and stabilizing agent. For this purpose, a different amount of Ag was doped to CuO and based upon degradation of MB, 2.7 mol % Ag–CuO was found more active catalyst as compared to other synthesized Ag–CuO nanocomposite. Here, SEM images exhibit spherical and rod-shaped particles before doping while some sort of softness and formation of sponge-like porous architecture was observed after doping. The HRTEM images showed that Ag is evenly doped on the CuO surface and it gives average crystallite size in the range of 30–90 nm. As a result, the Ag–CuO nanocomposite was found an efficient catalyst as it degrades 97% MB in an aqueous solution. In addition, ammonia was detected chemically even at very low concentrations. The high photocatalytic performance can be attributed to the synergetic coupling of Ag and CuO due to exceptional hollow hierarchical morphology that facilitates the proficient transportation and separation of the photo-generated charges during reaction hence boosting the photocatalytic performance.

## Data Availability

All data generated or analyzed during this study are included in this published article.
